# Supplementary material for: NGSpop: A desktop software that supports population studies by identifying sequence variations from next-generation sequencing data
Source: PLoS One. 2022 Nov 17;17(11):e0260908. doi: 10.1371/journal.pone.0260908 (PMC9671411; doi:10.1371/journal.pone.0260908)
Supplement: S1 File — (DOCX) [file pone.0260908.s001.docx]

# Availability and requirements

# Project name: NGSpop

# Project home page: https://sourceforge.net/projects/ngspop/

# Operating system(s): Linux

# Programming language: JavaFX

# Other requirements: All Perl libraries are listed in Supplementary.

# License: GNU General Public License

# Any restrictions to use by non-academics: license needed.

It is needed to install for running NGSpop.

1. Recent version of Strawberry Perl
2. Heap::Simple::XS module (prepare-refseqs.pl)

perl -MCPAN -e ‘force install Heap::Simple::XS‘

or cpan –f Heap::Simple::XS (Please, ignore some errors)

1. Devel::Size module and PerlIO::gzip module (flatfile-to-json.pl)

perl -MCPAN -e 'install Devel::Size‘

perl -MCPAN -e 'install PerlIO::gzip'

1. sudo apt install vcftools
